# Refractive Errors Affect the Vividness of Visual Mental Images

**DOI:** 10.1371/journal.pone.0065161

**Published:** 2013-06-05

**Authors:** Liana Palermo, Raffaella Nori, Laura Piccardi, Fabrizio Zeri, Antonio Babino, Fiorella Giusberti, Cecilia Guariglia

**Affiliations:** 1 Dipartimento di Psicologia, Sapienza Università di Roma, Rome, Italy; 2 Sezione di Neuropsicologia, Istituto Di Ricovero e Cura a Carattere Scientifico Fondazione Santa Lucia, Rome, Italy; 3 Dipartimento di Psicologia, Università degli Studi di Bologna, Bologna, Italy; 4 Dipartimento di Scienze della Salute, Università degli Studi dell'Aquila, Coppito, Italy; 5 CdL in Ottica e Optometria, Facoltà di Matematica, Fisica e Scienze Naturali– Università degli Studi Roma TRE, Rome, Italy; 6 Casa di cura “Tortorella” Salerno, Italy; University of Rome, Italy

## Abstract

The hypothesis that visual perception and mental imagery are equivalent has never been explored in individuals with vision defects not preventing the visual perception of the world, such as refractive errors. Refractive error (i.e., myopia, hyperopia or astigmatism) is a condition where the refracting system of the eye fails to focus objects sharply on the retina. As a consequence refractive errors cause blurred vision.

We subdivided 84 individuals according to their spherical equivalent refraction into Emmetropes (control individuals without refractive errors) and Ametropes (individuals with refractive errors). Participants performed a vividness task and completed a questionnaire that explored their cognitive style of thinking before their vision was checked by an ophthalmologist. Although results showed that Ametropes had less vivid mental images than Emmetropes this did not affect the development of their cognitive style of thinking; in fact, Ametropes were able to use both verbal and visual strategies to acquire and retrieve information. Present data are consistent with the hypothesis of equivalence between imagery and perception.

## Introduction

Visual mental imagery, accompanied by the experience of “seeing with the mind's eye”, occurs when a visual representation is present but the stimulus is not actually being viewed [1], [2]. The phenomenological similarity between visual imagery and visual perception has been noted since the time of the Greek philosophers and has been experimentally investigated since the pioneering Perky's study [3]. Following the cognitive revolution, “analog” theories sustained that both visual mental imagery and visual perception share numerous common processes [4]. Many behavioural investigations support this point of view. For example, some studies have shown that people spend more time scanning farther distances in their visual mental images than scanning nearer ones [5]–[7] and that the time necessary to mentally rotate objects is directly proportional to the angular degree of rotation [8]. It has also been demonstrated that visual imagery interferes with vision [9] and that eye movements during imagery are similar to those during perception [10]. Moreover, neuroimaging techniques have disclosed a substantial overlap in neural activations during visual mental imagery and visual perception [4]. Furthermore, it is noteworthy that the execution of visual mental imagery tasks also activates the primary visual cortex [2]. For instance, imagining a visual scene activates the visual cortex even when the eyes are closed [11], and functional deactivation of the visual cortex degrades the quality of internally generated images [12]. At a deeper level of analysis, a study by Kreiman and co-workers [13] showed the existence of single neurons in the human temporal lobe that selectively alter their firing rates depending on the nature of the stimulus not only when it is perceived but also when it is imagined. The authors suggested that the firing of these neurons might represent a correlate of the percept common to vision and imagery.

The hypothesis of equivalence between imagery and perception has been formalized in a cognitive model in which a single structure, that is, the *visual buffer*, is used to hold visual percepts and images that are internally generated [14], [15]. As a consequence of this supposed equivalence, any visuo-perceptual deficit could be associated with a corresponding deficit in visual mental imagery. Indeed, in the literature many patients are reported who have deficits in both the visual perception and the visual mental imagery domain (for a review see [16], [17]). For example Levine [18] described a patient with visual agnosia and prosopagnosia and difficulties in imagery tasks (especially in imagining faces). Goldenberg [19] reported another patient with a left temporo-occipital lesion that was affected by alexia, color agnosia, visual agnosia and unable to mentally generate the colour and the shape of objects. However patients with selective deficits have also been described [16], [17]. For example, Farah and colleagues [20] described a patient who was unable to generate images from memory (as shown by his poor descriptions and drawings) but showed no visual perception deficits. Instead, Bartolomeo and co-workers [21] described a patient with agnosia and prosopagnosia who had good mental imagery abilities. Moreover, some neuroimaging studies failed to find the same neuronal activation during mental imagery and visual perception and, above all, did not report neuronal activation in the primary visual cortex [22]–[24], like some of the previously mentioned studies [11], [12]. On the basis of these data, we can only say that whether visual mental imagery is underpinned by the same neuronal mechanisms as visual perception is still a matter of debate.

A direct way of studying the link between vision and mental imagery is to assess visual imagery skills in patients with peripheral (eye or optic nerve damage) or central (optic tract or visual cortical areas) visual disorders [25]. Although many studies in the literature investigated the visual imagery skills of patients with visual central disorders [16], [17], few studies were carried out in populations of individuals with peripheral visual disorders [26]–[30]; also, in most of the latter studies the experimental groups included only blind participants or participants with a severe vision loss. Although studying blind individuals can help clarify more general imagery capacity in sensory modalities other than vision, it is not useful for analyzing the specific visual mental imagery process. Indeed, several evidences suggest that blind individuals solve visuo-imagery tasks by relying on experience in other sensory domains. It is likely that blind individuals compensate for the lack of vision by enhancing their auditory capacities [31], and by developing conceptual networks with more acoustic and tactile nodes [32]. According to Kosslyn [15] different kinds of mental images might be formed (i.e., motor images, auditory images and visual images) and, to our knowledge, until now studies evaluating patients with peripheral visual disorders analyzed something defined as haptic representations or pure spatial representations without visual content [33]. Therefore, a more direct way of studying visual mental imagery capacity is to test a population of individuals with peripheral visual deficits that are not so severe as to prevent them from perceiving the world by means of vision, such as those with refractive eye defects. Refractive errors (e.g., myopia, hyperopia or astigmatism) are caused by the eye's inability to correctly focus objects on the retina. As a consequence refractive errors cause blurred vision.

According to the theory that visual perception and visual mental imagery are equivalent, vision defects of this kind, even if corrected by lenses, should affect the vividness of visual mental images. In particular, individuals with refractive errors (Ametropes), as a consequence of their blurred vision, should generate visual mental image less vivid and out-of-focus than individuals without refractive errors (Emmetropes that is subjects who have normal vision without need of any correction). But, if visual perception and visual mental imagery do not share the same neural substrates we should fail to find differences in the quality of visual images between individuals with and without vision defects. In order to evaluate these different possibilities, we used a modified version of the Vividness Task [34], [35] to assess the vividness of visual mental images in a group of participants with refractive errors (Ametropes) and a group without refractive defects (Emmetropes). Marks [36] suggested that the vividness of a mental image refers both to the “luminosity and clarity of mental imagery” and to how much it approximates to an actual percept. Moreover, Cornoldi and co-workers [37] suggested that the vividness of an image is also defined by colour, details, shape and borders. As previous studies demonstrated a wide distribution of vividness of visual images among individuals using both subjective and objective measures [38]–[43], the present study could contribute to clarifying whether this variability emerged only because individuals with refractive vision defects (even corrected by lenses) were included in the study samples.

Finally, according to the theory of equivalence, vision defects might also affect an individual's cognitive style of thinking [44]–[46], which can be verbal (verbalizer) or visuo-imaginative (visualizer) depending on the strategy used to acquire and retrieve information [47], [48].

Basing on the theory of equivalence the hypothesis may be drawn that if the vividness of mental images is lower in people with vision defects, they might tend to adopt a verbal thinking style rather than a visual one. This hypothesis is supported by the evidence that in some tasks blind people perform as well as control participants when they adopt a verbal strategy, but more poorly if they adopt an imagery strategy [49]–[51], suggesting that a vision deficit can affect the use of specific imagery strategy to solve a task. On the other hand, Green and Schroeder [52] found correlation between being a verbalizer and the performance in verbal tasks, but not significant correlation between being a visualizer and the performance in visuo-spatial tasks. Similarly Lean & Clements [53] have found that visualizers' performance in spatial ability tests did not differ from that of verbalizers, indicating that some individuals who preferred to verbally process information outperform visualizer individuals on visuo-spatial tasks. However, more recent studies [54], [55] suggest the validity of the construct by demonstrating that while verbalizers tended to be a homogeneous group with an intermediate level of spatial ability, there are two kinds of visualizers, one with high spatial ability and another with low spatial ability. To disentangle this point, we investigated the cognitive style of thinking (i.e. verbalizers vs. visualizers) and its relation with the vividness of visual mental images in Ametropic subjects (i.e., people affected by visual defects due to refractive errors) and Emmetropic subjects (i.e., people without visual defects).

## Methods

### Ethics Statement

The study was approved by the local Ethics Committee of Santa Lucia Foundation in Rome and all participants gave their written informed consent according to the 1964 Declaration of Helsinki. Data are fully available upon request to the corresponding author.

### Participants

We recruited 84 healthy, non presbiopic participants (50 females, 34 males; mean age 25.9±7.2) who had no history of neurological or psychiatric disorders and who were naïve to the purposes of the experiment from individuals who had had their eyesight checked in two different practices. Information about each participant's optical correction was recorded and the Spherical Equivalent Refraction (SER), that is, the sum of the sphere value plus the cylinder value (with negative sign) divided by 2 [SER = sphere+(cyl/2)] was calculated for each eye.

Based on this calculation, each participant was assigned to one of two different groups: subjects with a full normal vision or *Emmetropes*, if the optical correction in both eyes had an SER between +0.50 Diopters and −0.25 Diopters and a cylinder value no higher than −0.25 Diopters; subjects with defective vision or *Ametropes*, if the optical correction was greater than in the emmetropic condition in both eyes. The final sample included 34 control participants without refractive errors (Emmetropes, EP; 18 women and 16 men; mean age = 23.71 yrs, S.D. = 6.24 yrs; mean education = 14.03 yrs, S.D. = 2.35 yrs) and 50 participants with refractive errors (Ametropes, AP: 32 women and 18 men; mean age = 26.94 yrs, S.D. = 6.75 yrs; mean education = 14.1 yrs, S.D. = 2.34 yrs). The mean SER of the AP group was −3.14±3.6 Diopters and −3.25±3.7 Diopters for the right and left eye, respectively.

### Materials

#### Verbalizer-Visualizer Questionnaire (VVQ) [48]

This is a self-administered questionnaire consisting of 15 true-false items selected from a longer questionnaire proposed by Paivio [47], who had developed an 86-item Way of Thinking Questionnaire, and utilizes a true/false response format. The VVQ investigates consistencies and preferences in processing visual versus verbal information and classifies individuals as either visualizers (also called imagers), who rely primarily on imagery when attempting to perform cognitive tasks, or verbalizers, who rely primarily on verbal-analytical strategies [56]. Eight items tapping visual processing and seven tapping verbal processing are contained in the scale. Statements about verbal thinking include, for example, “I enjoy work that requires the use of words” and statements for visual thinking “My thinking often consists of mental pictures or images”.

The items are keyed to visualizers: so each response “true” at the eight visual items and each response “false” at the seven verbal items indicates a preference for the visual mode (maximum score = 15) whereas a low score indicates a verbalizing (verbal) (minimum score = 0) way of thinking. The VVQ is not affected by social desirability set responding; furthermore, it has an acceptable degree of test-retest reliability and differentiates between the two extreme groups on vocabulary and imagery tests. It has also demonstrated good construct validity: Kirby and co-workers [57], for example, reviled that the verbalizer score correlated with verbal proficiency (e.g., test of vocabulary, verbal reasoning, and analogies), and the visualize score was correlated with spatial visualization ability. Moreover, as Jonassen and Grabowski [58] reported, up to now it is “the primary instrument used” in research concerning the visualizer–verbalizer dimension (p. 193).

#### Vividness Task

(VT; a modified version of [34]; used previously in [35]). This task was developed to test the vividness of mental images by asking the participant to imagine a common object (i.e., a bottle, an apple, a lamp). In particular, in this context, following D'Angiulli and Reeves [59], vividness is defined as “*a construct expressing the self-rated degree of richness, amount of detail and clarity of a mental image, as compared to the experience of actual seeing*”.

**Figure 1:**
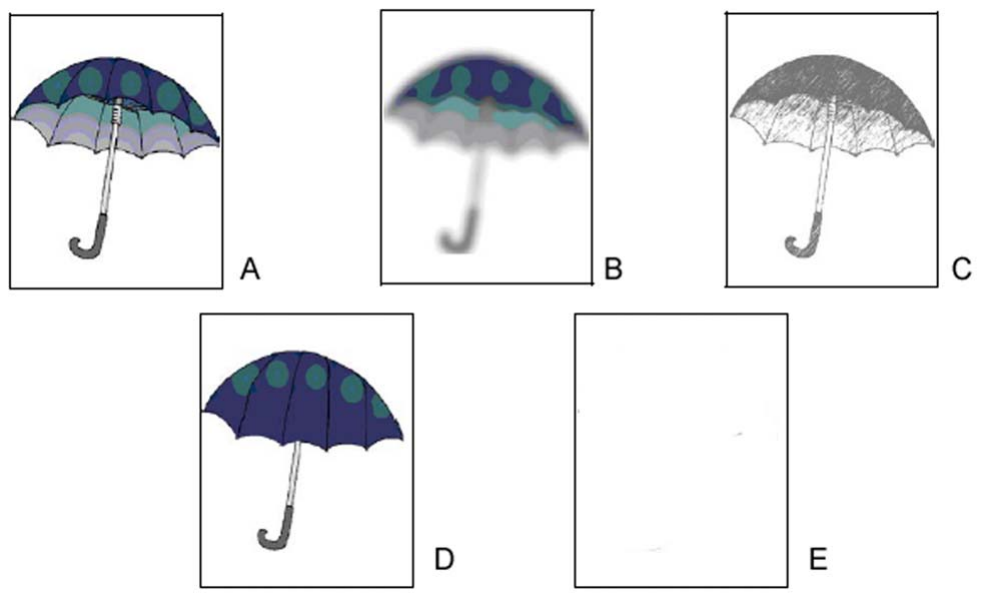
Example of Vividness Task items. Out of five pictures, participants had to select: A: a perfect 3D figure; B: an out-of-focus figure; C: a black-and-white figure; D: a 2D figure; E: no figure.

The participant is asked to image a specific object with closed eye and than to judge the vividness of his mental image on a Likert scale ranging from 1 (low vividness) to 7 (good vividness). Then, the participant was required to choose among five cards which one depicted a figure similar to his mental image. The five cards showed the same object depicted with different degrees of vividness (see figure 1 for an example): a perfect 3D figure (A), an out-of-focus figure (B), a black-and-white figure (C), a 2D figure (D), and no figure (E). B, C and D cards did not represent a continuum along A and E, since a specific loss of information is represented in each of them; namely in card B the picture lack of focus, in C of colour and in D of depth. The task consists of 20 items.

For the VT-Likert scale the maximum score was 140. The frequency with which each participant selected a specific kind of card (A, B, C, D, E), that is how many times each participant selected the A, B, C, D, or E cards, was recorded. For example a participant that imaged objects in a very vivid way would choose 20 times the card A and 0 times the other cards, achieving the following score: A = 20; B = 0; C = 0; D = 0; E = 0.

#### Visual assessment

The optical prescription following objective and subjective refraction was recorded for each participant in the form of a sphere, a cylinder (in negative values) and axes. Monocular best corrected visual acuity (BCVA) in ametropes and unaided visual acuity (UVA) in emmetropes were assessed: high contrast visual acuities were measured at a distance of 5 meters using an optotype projector (CSO; Florence, Italy), according to the Bailey-Lovie principles. Scoring was attributed using a letter-by-letter criterion in logMAR [60].

No significant difference was found between BCVA in Ametropic group and UVA in Emmetropic group.

### Procedure

Before the eye test, participants completed the VVQ and the VT without a time limit in a quiet, well-lit room. When asked to imagine the object in the VT, participants had to close their eyes to focus their attention on the visual imagery task. The administration order of the VVQ and the VT was counterbalanced across participants.

## Results

We performed a Mann-Whitney test to determine whether there were any differences among groups (EP and AP) in the total scores of the VT-Likert scale and the VVQ. The Mann-Whitney test showed no significant differences between EP and AP in the vividness of their mental images (U = 110.5; p = n.s.) or in their visual/verbal style (U = 635.5; p = n.s.).

**Figure 2:**
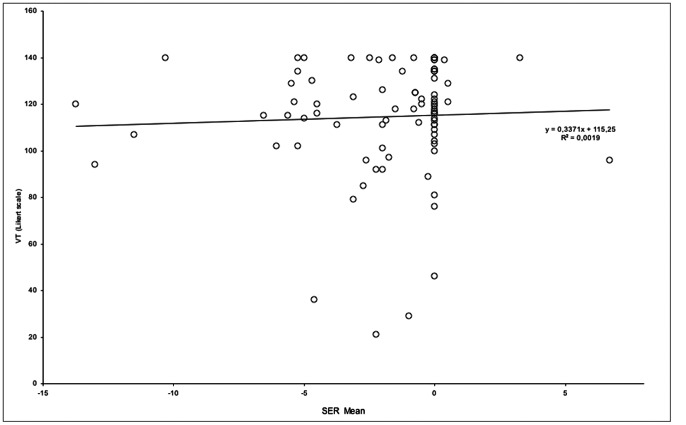
Scatterplot of the SER value (mean of both eyes) and VT Likert scale score.

**Figure 3:**
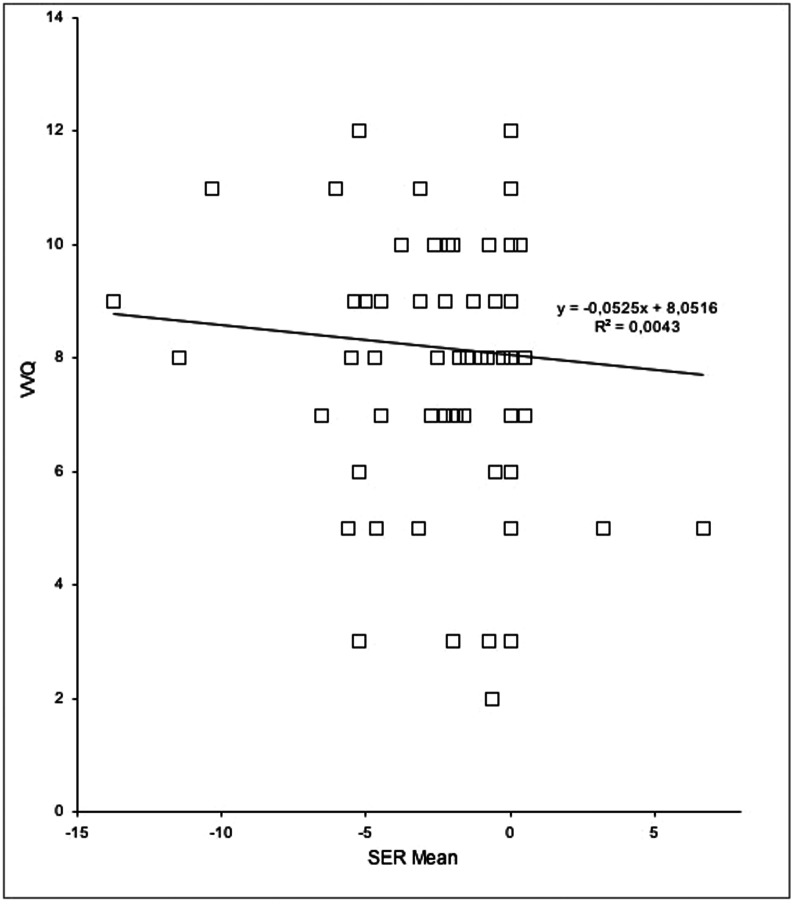
Scatterplot of the SER value (mean of both eyes) and VVQ score.

Moreover, to determine whether a correlation was present between ability to imagine and severity of refractive error, we performed Spearman correlations between the SER value (the mean of both eyes) and the total score obtained on the VT-Likert scale and the VVQ. We found no significant correlations between SER value and VT scores (r = 0.12; p = n.s.) or between SER value and VVQ scores (r = 0.036; p = n.s.). See figure 2 and figure 3.

We also performed a Chi-square analysis to evaluate the differences between the EP and AP groups in choosing a picture that corresponded to the generated image, in other words, which VT card (A, B, C, D and E) had been chosen as representing the participants' mental images better. Chi-square analysis on frequencies with which each cards was chosen by EP and AP showed a significant difference between the two groups in choosing the VT cards (Chi-Square = 39.4; df = 4 p<.005). Analysis of the Standardised Residual showed that compared with the critical value (±1.96), the B and C cards were selected with significantly lower frequency than expected in the EP group (B card: −3.56; C card: −2.75), and with significantly higher frequency than expected in the AP group (B card: 2.93; C card: 2.27). This last result was confirmed also performing a Mann-Whitney test, which showed no differences between AP and EP in choosing A (U = 695.5, p = n.s.), D (U = 783.5, p = n.s.) and E (U = 843, p = n.s.) cards, but statistically significant differences in choosing B (U = 654.50, p<.05) and C (U = 611, p<.05) cards.

**Figure 4:**
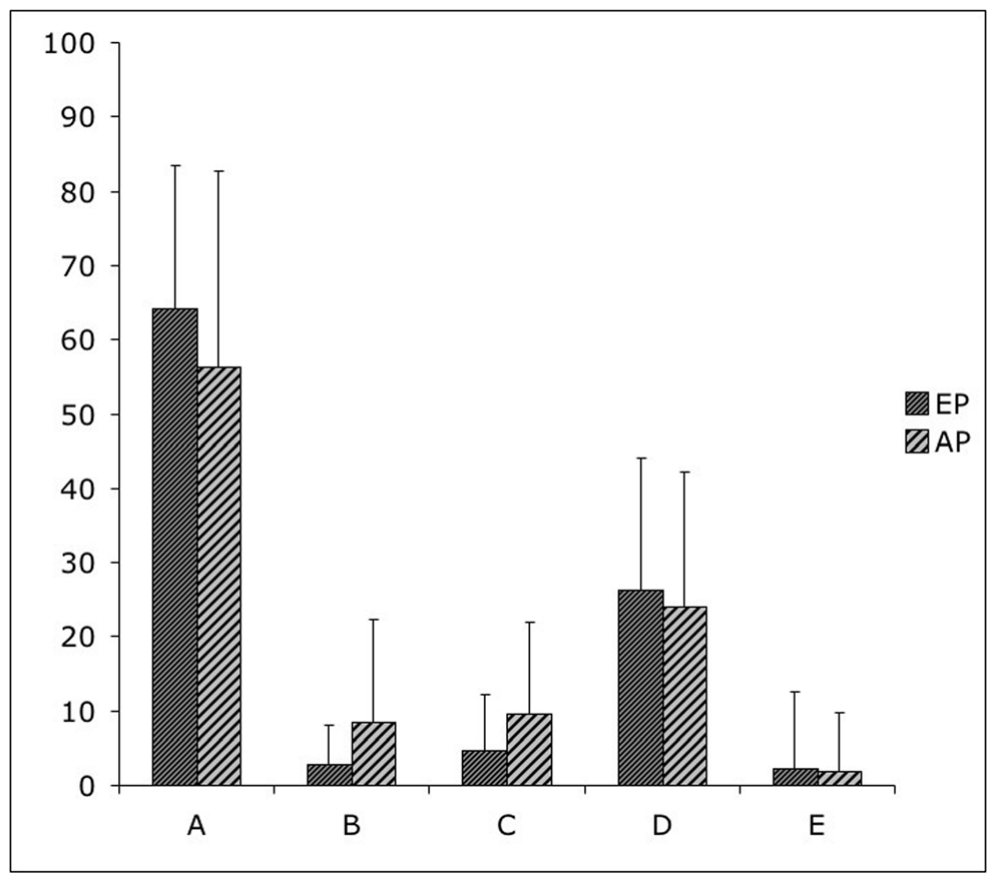
Percentage of the card selection at the Vividness Task for EP and AP groups. EP = Emmetropics; AP = Ametropics; A: a perfect 3D figure; B: an out-of-focus figure; C: a black-and-white figure; D: a 2D figure; E: no figure.

The percentage of the card selection for each group is presented in figure 4.

## Discussion

To our knowledge, this is the first study investigating the link between vision and visual mental imagery in individuals with peripheral vision defects who are still able to perceive stimuli. Up until now, studies on the role of peripheral vision deficits in mental imagery have explored the ability of creating and transforming mental images in individuals with acquired or congenital blindness. In these individuals, however, the building of a mental image is not mediated by visual inputs but by haptic, auditory or olfactory inputs and therefore it is difficult to compare in a direct way visual perception and visual mental imagery in this population. Instead, the correspondence between visual perception and visual imagery is primarily supported by results of individuals with central vision deficits. For example, Farah and colleagues [61] demonstrated a strict analogy between the perceptual visual field and the visual angle of the “mind's eye” in a patient submitted to unilateral occipital lobectomy who, after surgery, showed a reduction of the horizontal extent of both the contralateral visual field and the maximum size of mental images.

In the present study, we attempted to directly compare quality of perception and quality of imagery by analyzing the vividness of mental images in individuals with refractive vision defects (such as myopia). We hypothesized that the presence of a visual defect, even if corrected by lenses, would correspond to an analogous defect in visual imagery. In other words, the “blurring” of perception due to myopia or astigmatism would result in less vivid mental images. In fact, our results show that ametropic individuals tend to imagine objects as out-of-focus or without colour more often than control individuals without vision defects (Emmetropes). The fact that ametropic participants may generate out-of-focus images is consistent with their vision defects, in other words the “blurring”, frequently experienced by those people when the visual defect is not corrected, can be equivalent when they have to mentally image an objects. Also their choice of black and white images may be linked to the poor definition of the images; indeed, in defining “skeletal” mental images Kosslyn [14] defined them as images with low resolution, in which details such as colour and texture may be not represented. Alternatively, it may be hypothesized that the absence of colour in some images may be related to specific subclinical alterations in the perception of colours presented in some types of refractive errors. Thyagarajan and co-workers [62], indeed, found a slight effect of refractive blur induced in normal subjects using convex spherical lenses on colour vision performance. What remains to be analysed is if different types of refractive errors or different severities of refractive errors correspond to different types of visual mental imagery vividness. Also it would be interesting to understand if the effect of refractive errors on mental imagery is just limited to the vividness of mental images.

It is noteworthy that in our sample all the ametropes generated less vivid images despite the fact that they had a corrected vision. Therefore, one may wonder why individuals who wear glasses or contact lenses, and have visual acuity comparable to that of emmetropic participants, imagine objects less vividly than they perceive them. One explanation is that the deficit of most participants in the experimental group had not been corrected by glasses or contact lens for a certain period of their life, because they were too young to understand that they had a deficit or because the deficit did not hinder their daily life activities. Most ametropic individuals have defects that tend to worsen with time and often there is a variable, unascertainable delay between the onset of deficit worsening and the moment when the individual recognizes it. Therefore, many of the experimental participants may have experienced one or more time intervals of uncorrected or partially corrected vision that affected their imagery vividness. Indeed, the ametropic participants were recruited in clinical offices where they had gone for examination because their eyesight had worsened. One may also wonder how long visual defects have to last before they affect visual imagery and whether long-lasting correction of vision might improve the vividness of mental imagery. It is noteworthy, however, that individuals with lower imagery vividness judge their own vividness as very good and that no differences were found between the ametropic and emmetropic individuals in judging their own imagery vividness. This lack of awareness of vividness in ametropes needs to be discussed. First, it is difficult for a person to obtain feedback about the quality of their visual mental images. Thus, people with vision defects can acknowledge their perceptual deficits by comparing their ability to perceive in daily life with that of people without vision defects. For example, myopic individuals recognize the presence of a vision defect when they have difficulty in perceiving a distant object indicated by another individual. But, it is less likely that people who are unable to imagine an object in detail will be able to compare their own mental images with those of people who have high level visual imagery skills. Therefore, in the absence of objective feedback people are persuaded that their own mental imagery skills are at the highest possible level of mental visualization.

However, it is interesting that Ametropes choose also the well-defined card in the Vividness Task (card A). Considering that people with myopia can see objects that are near but have difficulty seeing distant objects, we can speculate that when an object was imagined in the far space they choose an out-of-focus card, while when an object was imagined in the near space they choose a well-defined card. The reverse would happen with participants with hyperopia who can see objects that are far but have difficulty seeing close objects. Unfortunately we can not verify this hypothesis because most, if not all, of the objects of our Vividness Task can be imagined in both far or near space (for example one could image a chair close or very far to himself/herself). Further studies, explicitly comparing the vividness of images generated at different distances (e.g., a clock face on the wrist or on the top of a bell tower) in individuals suffering from different types of refractive errors, are necessary to test this interpretation.

A second comment concerns the possible bias in studies of mental imagery that adopt self-report to control imagery vividness. Studies on visual mental imagery vividness report a wide distribution of vividness ability. This might just be due to the presence of individuals with peripheral visual defects in the sample that were not considered in these previous studies, because vision defects were corrected by lenses. According to the theory of equivalence between imagery and perception, a deficit in correctly perceiving the environment results in lower vividness of internal visual images. In fact, participants with visual defects tend to select out-of-focus and black and white pictures, suggesting that the pictorial features of their mental images are less vivid than those of participants with normal vision. This suggests that the investigation procedure is crucial in determining the presence/absence of differences among groups. Indeed, when people are asked to imagine an object and mentally evaluate it on an abstract scale of values (very good vs. very poor), they are less realistic in judging their ability than when they are asked to imagine an object and evaluate it by comparing their mental image with alternative real pictures. When using the latter procedure, our data fit with studies that found deficits in both the visual perception and the visual mental imagery domains in brain-damaged patients (for a review see [16], [17]) and with neuroimaging reports that showed a substantial overlap between neuronal areas involved in both vision and visual mental imagery [11], [12].

Another issue concerns studies on mental imagery skills that adopt self-evaluations of image vividness to select participants. As we discussed earlier in this paper, people may not be aware that their images are less vivid than they could be. Therefore, if participant selection is based on verbal reports of ability to imagine objects, there is a risk of including participants with very different degrees of visual imagery skills in the experimental group. Thus, it is possible that the great variability reported in visual mental imagery is at least partially due to the presence of people in the observed population who are suffering from visual defects and therefore with low imagery vividness.

A second aim of this study was to assess whether the presence of a visual defect, implying low levels of imagery vividness, might influence cognitive style of thinking. We observed that in our sample the preference for verbal or visual strategies to acquire and retrieve information is not related to the presence/absence of visual defects. This suggests that cognitive style of thinking is not influenced by the quality of vision, but that other individual differences may affect the cognitive style of thinking (e.g., differences in memory skills for verbal and non-verbal material, the level of linguistic skills, educational factors, etc.). In present study we just analyzed the possible presence of correlations between the subjective preference to verbalize or the visualize for information processing and the presence of refractive errors, without evaluating if the cognitive style is related to individual differences in the level of proficiency in verbal and visuo-spatial activities. Thus, the present study does not allow to draw any conclusion about the relation between a given cognitive style and proficiency in a type of task (i.e., the effect of being a visualizer on the proficiency in visuo-spatial activities), but just underlines that the concomitant presence of refractive errors and less vivid visual imagery does not predict the individual preference for using verbal coding of information. Present data also suggest the possibility that the strategy used for processing information should mainly depend from the type of task or problem to be solved. For instance, to recognize a point of reference (i.e. a certain building) in the environment it is more useful to adopt a visual strategy, which only requires remembering one item, than a verbal one, which requires remembering a long string of information. Thus, individuals use a visual thinking cognitive style because it is more economical even if their mental image is not as vivid. The recognition of a certain building among others will be not invalidated by the fact that the mental image does not contain colours or is out-of-focus if it contains all the relevant features allowing for its identification.

In conclusion, the present results do not support the hypothesis that verbalizer/visualizer cognitive style depends upon the level of visual abilities but support the perception-imagery equivalence hypothesis, demonstrating that equivalence does not just refer to the cortical level of perceptual systems but also to more peripheral ones. A novel result was the observation of ametropic individuals' low ability to verbally judge image vividness, which suggests the need to adopt other types of subjective assessment to evaluate the quality of mental imagery.

Finally, although subjective rating is a standard method used in psychology and psychophysics, further studies evaluating the vividness of mental images with more objective measures, for example, using neuroimaging techniques [43] in individuals with vision defects could shed more light on this topic.
